# Ammonia-Mediated Bulk Synthesis of Layered Nitride Materials

**DOI:** 10.3390/ma19142998

**Published:** 2026-07-11

**Authors:** Masashi Tanaka, Takuma Hirao

**Affiliations:** Graduate School of Engineering, Kyushu Institute of Technology, Kitakyushu 804-8550, Japan

**Keywords:** ammonia-mediated synthesis, ammonia chemistry, layered nitride materials, transition metal nitride halides, superconductivity

## Abstract

Ammonia provides a versatile platform for the synthesis and chemical modification of layered nitride materials. Among these, layered nitride halides of the *M*N*X* family have attracted considerable attention because of their superconductivity upon electron doping and their rich structural chemistry. Many of these compounds, including both α- and β-type phases, are synthesised via ammonia-mediated reactions, highlighting the central role of NH_3_ chemistry in this class of materials. In this review, we summarise ammonia-mediated synthesis routes and reaction environments for layered nitride halides and related nitride materials, with particular emphasis on bulk synthesis strategies. As a case study, we discuss the synthesis and electron-doped superconductivity of TiNI, a less explored member of the *M*N*X* family. We further highlight recent developments in ammonia-driven reactions that enable the formation and modification of nitride phases, including nitrogen-rich ZrN derived from layered precursors while retaining their morphological features. These examples demonstrate that ammonia-mediated processes provide a unified platform for controlling structure, composition, and electronic states across a wide range of reaction conditions.

## 1. Introduction

Ammonia (NH_3_) has long been recognised as a versatile reaction medium for the synthesis of inorganic nitrides and related materials, with early studies revealing its ability to transform metal halides into ammonia-derived solids–processes that can now be understood within the framework of ammonolysis chemistry [1,2]. Depending on the reaction conditions, ammonia can act not only as a nitrogen source but also as a reactive chemical environment that promotes nitridation, topochemical transformations, and defect formation in solid-state materials [3]. Among the material systems accessible through such chemistry, layered nitride halides of the *M*N*X* family (*M* = Ti, Zr, Hf; *X* = Cl, Br, I) represent a particularly important class of materials because of their superconductivity and rich structural chemistry [1,2]. Previous studies have extensively reviewed the structural, electronic, and superconducting properties of *M*N*X* compounds [4,5].

**Figure 1:**
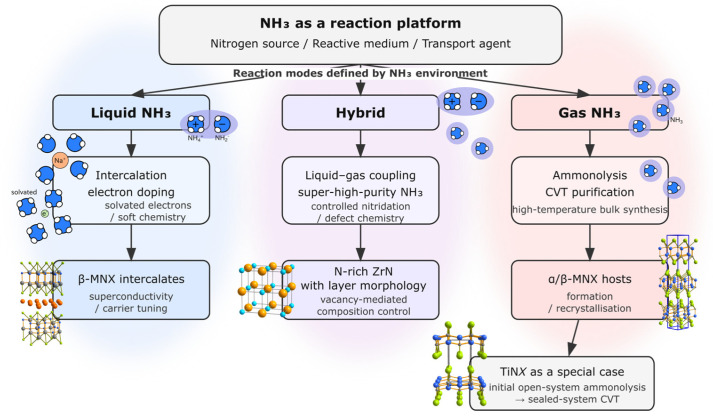
Conceptual overview of ammonia-mediated synthesis of layered nitride materials discussed in this review (This work). NH_3_ serves multiple roles as a nitrogen source, reactive medium, and transport agent, enabling a range of synthetic pathways depending on its physical state. Gas-phase reactions enable ammonolysis and chemical vapour transport (CVT) processes for the formation and purification of *M*N*X* compounds and related nitrides. Liquid ammonia enables low-temperature intercalation chemistry and electron doping, particularly for β-type structures. A hybrid approach, combining liquid and gas phases, provides a high-purity ammonia environment for controlled nitridation and defect engineering. These distinct reaction modes collectively determine the accessible structures and compositions of the resulting nitride materials. The species shown in the liquid and hybrid regions are schematic representations of ammonia-based reaction environments, including self-ionisation equilibria in liquid ammonia (e.g., NH_4_^+^/NH_2_^−^) and solvated electron species.

In contrast to previous property-focused reviews, the present review emphasises ammonia-mediated bulk synthesis as a unifying synthetic platform for layered nitride materials. In particular, we classify ammonia-based reactions into gas-phase, liquid-ammonia, and hybrid gas-liquid routes, and discuss how these environments enable both the synthesis and chemical modification of layered nitride halides and related nitride phases [3,6,7]. Other important topics, including thin-film fabrication, nanoscale materials processing, and detailed theoretical aspects, are beyond the scope of the present review and warrant separate discussion. This review adopts a perspective-style approach, incorporating selected case studies to highlight recent developments. The general concept of these ammonia-mediated synthesis routes is illustrated schematically in Figure 1.

## 2. Gas Phase Reactions

### 2.1. MNX Overview

Transition-metal nitride halides *M*N*X* (*M* = Ti, Zr, Hf; *X* = Cl, Br, I) were first reported in the 1960s by Juza and co-workers, who demonstrated that reactions between metal halides and ammonia can yield layered nitride halides. A variety of Ti- and Zr-based nitride halides were synthesised during this period [8,9,10,11].

**Figure 2:**
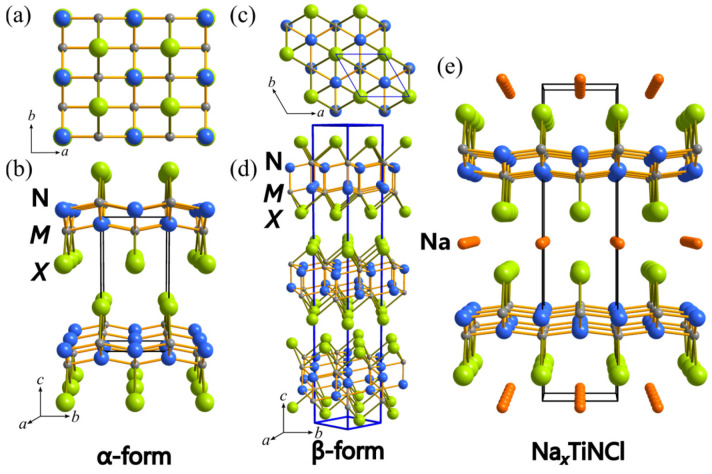
Schematic crystal structures of *M*N*X* layered nitride halides based on published structural data (This work): (**a**,**b**) α-form (FeOCl-type) and (**c**,**d**) β-form (SmSI-type). In both structures, covalently bonded *M*–N layers are separated by halogen layers, forming quasi-two-dimensional frameworks. The α-form structure consists of an orthogonal *M*N network, whereas the β-form structure features double honeycomb-like *M*N layers. (**e**) schematic side view of a representative sodium-intercalated TiNCl structure, illustrating layer expansion upon intercalation.

These compounds crystallise in two polymorphs, namely the α- and β-type structures, which adopt the FeOCl- and SmSI-type structures, respectively [3]. In both polymorphs, covalently bonded *M*–N layers are separated by halogen layers, resulting in a quasi-two-dimensional crystal and electronic structure. The α-type polymorph has an orthogonal *M*N layer network as shown in Figure 2a,b, whereas the β-type polymorph consists of double honeycomb-like *M*N layers as illustrated in Figure 2c,d.

A major breakthrough occurred when Yamanaka and co-workers discovered that electron doping via alkali-metal intercalation induces superconductivity in layered nitride halides [12,13,14,15]. Upon electron doping, the band-insulating *M*N*X* compounds become metallic and exhibit superconducting transition temperatures (*T*_c_s) exceeding 25 K in some cases [3,6,13]. Superconductivity can also be induced by carrier doping using an electronic double-layer transistor [16,17,18,19,20].

Subsequent studies have revealed a range of unusual superconducting properties, including the absence of a conventional isotope effect, large superconducting gap values (2Δ/*k*_B_*T*_c_, where Δ is the superconducting gap and *k*_B_ is the Boltzmann constant), and anomalous carrier-density dependence of *T*_c_, and references therein [21,22,23,24,25,26,27,28,29,30,31,32,33,34,35,36,37,38]. These observations suggest that the superconducting state in these materials cannot be fully explained within a conventional phonon-mediated Bardeen–Cooper–Schrieffer (BCS) framework [39].

Titanium nitride halides (TiN*X*) crystallise exclusively in the α-type structure. TiNCl exhibits superconductivity with transition temperatures of up to ~16 K upon electron doping through metal and/or organic intercalation [14,40,41]. A representative sodium-intercalated TiNCl structure is illustrated schematically in Figure 2e. However, α-type compounds are generally less favourable hosts for intercalation chemistry than β-type materials. In contrast to β-type compounds, which are relatively stable in air and even in acidic environments, α-type materials are highly sensitive to moisture and can readily decompose into binary *M*–N phases [42,43].

Despite these challenges, α-*M*N*X* systems have recently attracted renewed interest owing to their unique two-dimensional electronic structures and potential applications in energy and electronic devices [44,45,46,47,48,49,50,51,52]. However, many of these proposals are based on first-principles calculations, while experimental studies remain limited, although recent studies have begun to expand the available experimental data [30,53,54,55,56,57,58,59], largely due to the difficulties associated with synthesising both the parent α-type compounds and their electron-doped derivatives. This situation highlights the importance of revisiting synthetic strategies for *M*N*X* compounds, particularly those based on ammonia-mediated reactions.

### 2.2. Synthesis of MNX (M = Zr, Hf)

The synthesis of transition-metal nitride halides generally relies on ammonia-mediated reactions of metal halides. In a simplified form, the formation of *M*N*X* compounds can be represented by the reaction

*MX*_4_ + 4NH_3_ → *M*N*X* + 3NH_4_*X*

in which the metal formally retains its oxidation state.

In practice, however, the ammonolysis of metal halides proceeds through complex reaction pathways involving intermediate amide species and the formation of various by-products [4]. As a result, direct reactions between *MX*_4_ and NH_3_ typically yield poorly crystalline mixtures rather than phase-pure *M*N*X* compounds.

A key advance in *M*N*X* synthesis was the recognition that ammonium halides generated during ammonolysis can act as effective transport agents. Yamanaka and Ohashi demonstrated that NH_4_*X* species formed in situ mediate chemical vapour transport (CVT) of layered nitride halides, enabling recrystallisation under a temperature gradient and yielding highly crystalline samples suitable for structural studies [14].

This discovery established ammonia-mediated CVT as a key step in the preparation and purification of *M*N*X* compounds. Although the initial ammonolysis reaction typically produces mixtures of phases, the subsequent transport process enables purification and crystal growth of the nitride halide phase. Building on this concept, Yamanaka later developed an efficient synthetic route in which metal or metal hydride powders react directly with ammonium halides, NH_4_*X*, under ammonia-rich conditions [14]. Thus, ammonia-mediated synthesis of *M*N*X* compounds typically involves a two-step process consisting of an initial ammonolysis reaction followed by a closed-system CVT step for purification and crystal growth.

A representative example is the preparation of ZrNCl [3,60]. In this method, zirconium metal or zirconium hydride powders react with ammonium chloride in an open system at elevated temperatures (~650 °C) according to

Zr (or ZrH_2_) + NH_4_Cl → ZrNCl + 2 (or 3) H_2_.

The reaction is typically performed in a two-zone horizontal furnace in which ammonium chloride is placed in the lower-temperature region while the metal precursor is located in the higher-temperature region. Ammonium halide vapour is transported to the metal source, where nitridation occurs.

The resulting product can subsequently undergo purification by chemical vapour transport in a closed system mediated by NH_4_Cl (NH_3_ + HCl), allowing highly crystalline ZrNCl to recrystallise in the hotter region of the reaction tube [3]. In Zr- and Hf-based systems, both α- and β-type polymorphs can be obtained depending on the reaction conditions. In particular, the crystal structure can be controlled by the temperature gradient employed during CVT. For example, in the case of ZrNCl, the β-form phase is typically obtained at higher temperatures (e.g., 750–850 °C), whereas the α-form phase can be stabilised at lower temperatures (e.g., 390–490 °C). This tunability reflects the sensitivity of *M*N*X* formation to thermodynamic and kinetic conditions during ammonia-mediated reactions.

This strategy can be extended to other combinations of metals and halogens, and various *M*N*X* compounds can be synthesised by selecting appropriate metal powders and ammonium halides. The transport-assisted growth process is particularly effective for producing well-crystallised layered nitride halides suitable for structural and physical-property studies. A simplified one-step synthesis in a vacuum-sealed glass tube can also be employed for small-scale preparations of less than ~200 mg [3,13]. Because the entire reaction can be conducted in a closed system, this approach is particularly advantageous for ^15^N isotope substitution, as the nitrogen source can be precisely controlled.

While such polymorph control is well established for Zr- and Hf-based systems, the situation is markedly different for Ti-based compounds, as discussed in the following section.

### 2.3. Synthesis of Ti-Based (α-Form) MNX: TiNCl as a Representative Example

In contrast to Zr- and Hf-based systems, in which ammonia-mediated synthesis can be carried out entirely in a closed system, Ti-based nitride halides present significant synthetic challenges. For *M* = Ti, particularly in the case of TiNCl, whose preparation was first described in 1964 [11], the simplified synthetic route starting from the metal described above is not applicable [43]. Early attempts to synthesise TiNCl using simpler or less elaborate approaches have generally not yielded favourable results. For example, the direct halogenation of TiN resulted in the formation of oxychloride phases rather than the desired nitride chloride, as reported by Rudge and Arnall and later discussed by Hock and Knauff [61]. Subsequent approaches involving the direct nitridation of TiCl_4_ with active nitrogen [62] also failed to yield pure TiNCl, instead leading to chlorine-rich adducts, such as TiNCl·TiCl_4_ [61].

These unsuccessful attempts reflect the intrinsic reactivity and complexity of titanium chemistry. When titanium metal reacts with NH_4_Cl in a closed system, TiH_2_ is first formed at temperatures around 400 °C or lower. At higher temperatures where TiH_2_ decomposes, TiN is preferentially formed instead of TiNCl. Additionally, the corresponding metal halide, TiCl_4_, is a liquid at room temperature and pressure, further complicating reaction control under ammonolysis conditions. These factors indicate that the synthesis of TiNCl cannot be achieved entirely within a closed system. Instead, it requires a two-step process: an initial ammonolysis under flowing NH_3_ in an open system, followed by purification via CVT in a sealed system. This contrasts with the Zr- and Hf-based systems described above.

Because titanium tetrachloride reacts extremely readily with ammonia to form aminated titanium species, ammonolysis of TiCl_4_ provides a practical route to TiNCl. In this approach, ammonia gas is passed over TiCl_4_ in an open reaction system, leading to rapid conversion to a black solid due to the reaction’s highly exothermic nature. The high reactivity of TiCl_4_ results in the formation of various ammonia-containing intermediates [63,64], including adducts such as TiCl_4_·*x*NH_3_ and aminated species [65]. Upon further heating under ammonia-rich conditions, stepwise amination of TiCl_4_ proceeds to form TiCl(NH_2_)_3_ [65,66,67].

The reaction temperature must be carefully controlled around 400 °C under continuous flow of ammonia gas to promote TiNCl formation: lower temperatures favour ammonia complexes and aminated TiCl_4_ intermediates [65], whereas higher temperatures lead to decomposition into TiN [68,69]. During this process, ammonium chloride deposition is observed downstream of the reaction zone.

The resulting precursor mixture, containing TiNCl and related compounds, is subsequently subjected to CVT with a temperature gradient typically of 380–420 °C using NH_4_Cl in a sealed system, enabling purification and recrystallisation of TiNCl [70]. Once reduced to TiN, regeneration of TiNCl is difficult, requiring careful control of the reaction temperature within a narrow window slightly above 400 °C to obtain impurity-free samples.

The CVT process can be rationalised by a mechanism analogous to that proposed for ZrNCl [14] as described by the following reactions:

TiCl_4_(g) + NH_3_(g) ⟶ TiCl_3_(NH_2_) + HCl ⟶ TiNCl(s) + 3HCl(g) (>~400 °C).

In practice, NH_4_Cl is used as a source of HCl through thermal dissociation, leading to the overall reaction:

TiCl_3_(NH_2_) + NH_4_Cl ⟶ TiNCl(s) + NH_3_(g) + 3HCl(g).

The phase purity of the obtained TiNCl can be confirmed by Rietveld refinement and magnetisation measurements, typically showing negligible diamagnetic contributions from TiN impurities.

However, the requirement for an open ammonolysis step also complicates the preparation of ^15^N-substituted TiNCl, as it necessitates a continuous supply of isotopically labelled ammonia gas. This is particularly important for investigating isotope effects, which provide key insights into whether superconductivity is mediated by phonons, with nitrogen isotopes offering particular advantages due to their larger relative mass change and compatibility with NMR measurements. To address this limitation, an alternative synthetic approach employing a solid nitrogen source, NaNH_2_, has been proposed [55]. This method enables the entire synthesis to be carried out in a closed system, although ammonia-based routes remain the most widely used and effective for bulk synthesis.

### 2.4. Iodine Analogue: TiNI

As an illustrative example within the framework of ammonia-mediated synthesis, we briefly present selected results on TiNI to highlight the applicability of the synthetic strategy. Although TiNCl has been widely investigated as a representative α-type nitride halide, much less is known about the corresponding iodine analogue. In particular, given the synthetic challenges associated with Ti-based systems described above, TiNI provides a useful case study for examining how these limitations manifest in iodine-containing layered nitride halides. TiNI has been reported in early studies [11], but detailed investigations beyond basic structural characterisation remain limited. Even basic crystallographic details, such as reliable estimates of lattice parameters and their uncertainties, have remained scarce.

**Figure 3:**
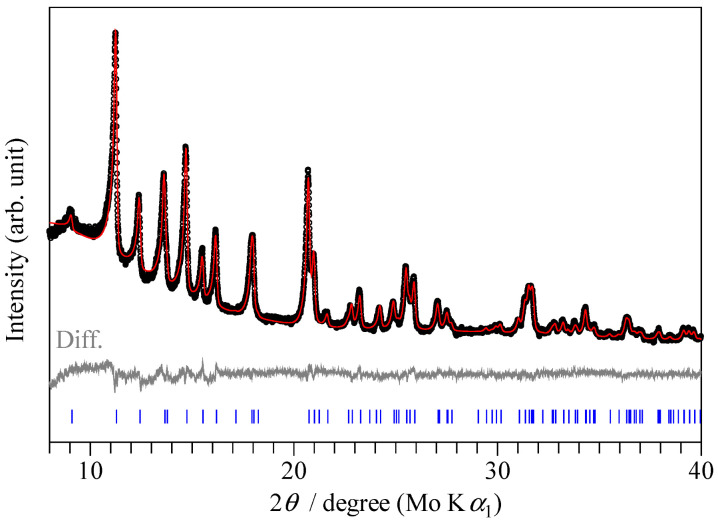
Rietveld refinement profile for TiNI. Open circles represent the observed diffraction data, the solid line indicates the calculated pattern, and the difference curve is shown in grey. Bragg reflection positions are marked in blue, confirming the formation of the α-type TiNI phase (This work).

The synthesis procedure follows the general strategy described for TiNCl, involving an initial ammonolysis of titanium tetraiodide (TiI_4_) followed by recrystallisation via CVT. Precursor formation typically proceeds at temperatures between 180 and 250 °C. The transport process is carried out with NH_4_I as a transport agent under a temperature gradient of 230–350 °C in a horizontal furnace. Under these conditions, plate-like TiNI crystals were obtained. The powder X-ray diffraction pattern of the product was analysed by Rietveld refinement (Figure 3), confirming the formation of the α-type TiNI phase. The refined structural parameters are summarised in Table 1 and Table 2. These data provide a basis for comparison with the chloride analogue and enable a more precise discussion of structural differences within the α-type series.

**Figure 4:**
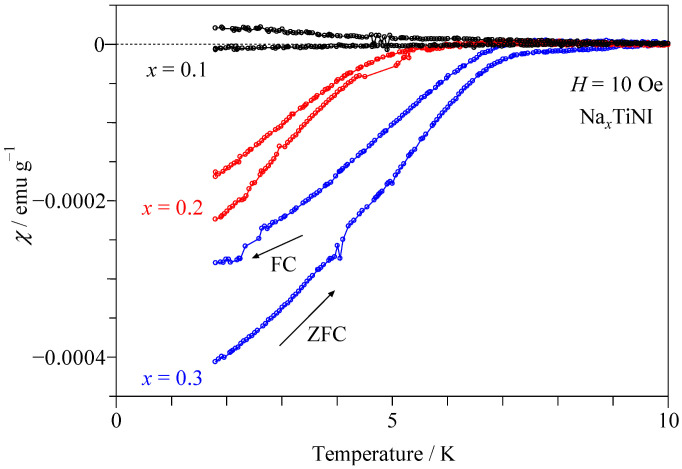
Temperature dependence of magnetic susceptibility for Na-intercalated TiNI with different carrier concentrations measured in the zero-field-cooling (ZFC) and field-cooling (FC) modes (This work).

Although no clear evidence of intercalation results for TiNI has been reported to date, superconductivity is expected to emerge upon electron doping. Preliminary measurements indicate that electron doping of TiNI induces superconducting behaviour with a *T*_c_ of approximately 7 K, as shown in Figure 4. The sodium intercalation was carried out using a Na-naphthalene solution in tetrahydrofuran (Na-Naph/THF) [56]. The superconducting volume fraction is relatively small, and no significant structural changes could be clearly resolved by powder X-ray diffraction, implying that the intercalation may be inhomogeneous or limited in extent. The observed superconductivity is unlikely to originate from TiN impurities, whose superconducting transition temperature is considerably lower. Further investigations are currently in progress and will be reported elsewhere.

Practical considerations. Gas-phase ammonolysis combined with CVT typically yields well-crystallised products, often as plate-like crystals suitable for structural and physical-property studies. The method generally requires several days to weeks and involves moderate experimental complexity, including sealed-tube handling and temperature-gradient control. Although usually limited to small-scale synthesis (tens to hundreds of milligrams), careful loading enables safe and reliable preparation of high-quality samples.

## 3. Liquid Ammonia Reactions

In addition to gas-phase reactions discussed above, ammonia can also serve as a condensed-phase reaction medium. In this case, liquid ammonia provides a chemically active solvent environment that enables reaction pathways distinct from those in the gas phase. Although liquid ammonia chemistry has been less explored for bulk synthesis of layered nitrides, several studies have demonstrated its usefulness in ammonolysis and intercalation reactions.

### 3.1. Ammonolysis Reactions in Liquid Ammonia

Liquid ammonia can serve not only as a reagent but also as a reactive solvent for ammonolysis reactions, enabling the formation of nitrogen-containing precursor species under relatively mild conditions. One early example involves the reaction of zirconium tetrachloride with liquid ammonia, which yields zirconium amide chloride species [71].

In this reaction, ZrCl_4_ undergoes partial ammonolysis, in which one of the Zr–Cl bonds is replaced by an amide group, producing zirconium amide chloride:

ZrCl_4_(g) + (*n* + 2)NH_3_(g) ⟶ ZrCl_3_(NH_2_)·*n*NH_3_ + NH_4_Cl.

**Figure 5:**
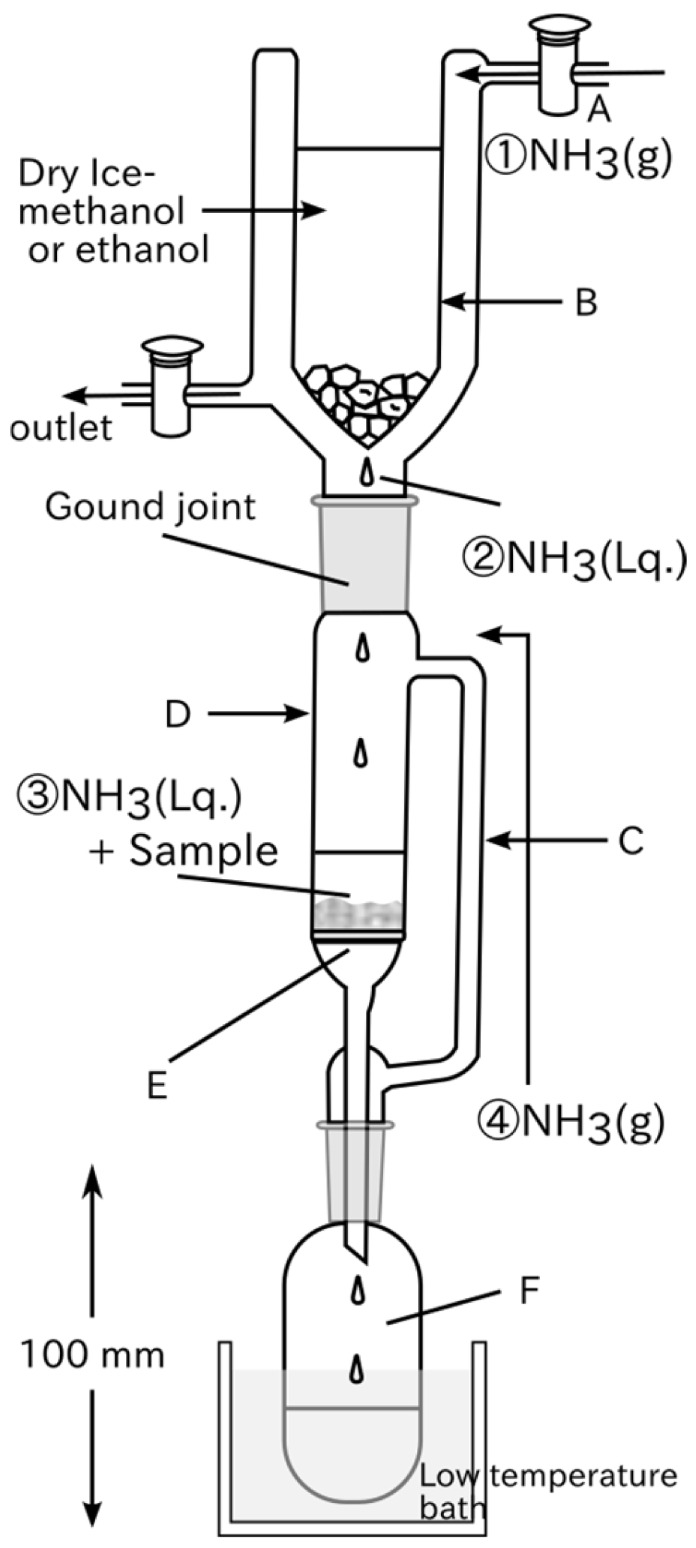
Schematic illustration of the liquid ammonia circulation apparatus based on a Soxhlet-type extractor reported in Ref. [71], with permission from Oxford University Press. A: valve, B: condenser, C: bypass line, D: reaction tube, E: glass filter, F: flask.

The ammonium chloride produced during the reaction can be removed by washing the solid product with liquid ammonia. To carry out this process efficiently, a glass apparatus (Figure 5) was designed to continuously circulate liquid ammonia through the reaction zone. In this setup, zirconium tetrachloride is placed on a glass filter (E) inside a reaction tube (D), which is connected to a condenser (B) cooled by a dry ice-alcohol mixture. Dry NH_3_ gas is introduced through valve (A) and condensed in the condenser.

The condensed liquid ammonia drips onto the ZrCl_4_, where ammonolysis occurs. The ammonium chloride formed during the reaction dissolves in the liquid ammonia and is transported to a collection flask (F) located below the reaction tube. Ammonia evaporating from the lower flask is returned to the condenser via a bypass line (C), where it is liquefied again, enabling continuous circulation of liquid ammonia. In this way, the apparatus operates similarly to a Soxhlet extraction system, continuously washing the reaction mixture with fresh liquid ammonia.

After the ammonolysis process is completed, the liquid ammonia is removed, and the system is purged with an inert gas. The zirconium amide chloride remains on the glass filter as a white solid. The resulting amide species can subsequently be converted into ZrNCl upon heating under a stream of ammonia gas. This example illustrates that liquid ammonia can function not only as a reagent but also as a reaction medium for precursor formation in the synthesis of layered nitride materials. In addition to such precursor chemistry, liquid ammonia also enables intercalation and reductive processes, as discussed in the following section.

### 3.2. Intercalation Reactions in Liquid Ammonia

Liquid ammonia is also widely used as a medium for intercalation reactions in layered nitride halides [6,72]. Alkali metals readily dissolve in liquid ammonia to form deep-blue metal-ammonia solutions containing solvated electrons, which act as powerful reducing agents. These highly reducing species facilitate electron doping of layered host materials, enabling intercalation reactions in the van der Waals gaps.

These solutions have been extensively employed to introduce alkali metals into layered *M*N*X* compounds. Electron doping through such intercalation reactions has been shown to induce superconductivity in β-type nitride halides, including ZrNCl and HfNCl. Liquid ammonia thus provides a convenient pathway for both electron doping and structural modification in layered nitride systems.

**Figure 6:**
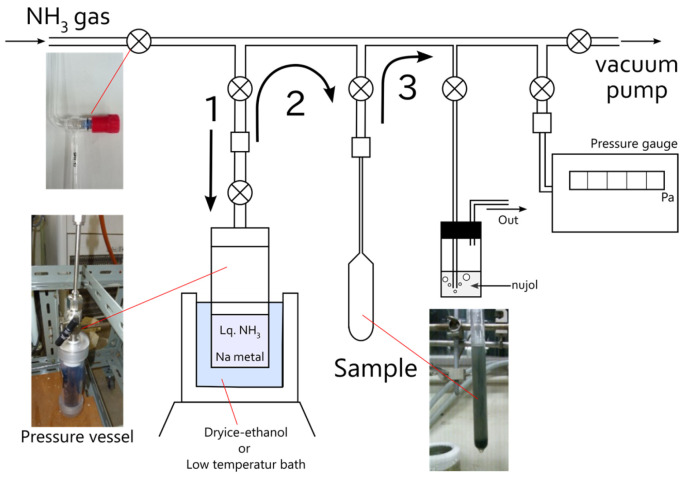
Schematic illustration of a liquid-ammonia intercalation apparatus with an ammonia reservoir and transfer system for handling purified liquid NH_3_ under controlled conditions (This work). Numbers indicate the sequence of operation.

A typical apparatus used for intercalation reactions in liquid ammonia is illustrated in Figure 6. In this system, ammonia gas is first condensed into a pressure vessel containing alkali metal, which serves to remove residual oxygen and moisture impurities. This provides a reservoir of purified liquid ammonia.

The reaction cell containing the host material is then cooled, and upon opening the valve, purified ammonia is transferred in the gas phase through the evacuated line and condensed in the cooled reaction vessel, forming a metal–ammonia solution. The sample is typically immersed in the solution for several hours at low temperature (e.g., in a dry-ice–ethanol bath). The characteristic deep-blue colour of the solution disappears upon completion of the intercalation reaction. After the reaction, the system is warmed to room temperature, and the ammonia is removed by evaporation followed by evacuation.

A representative example of intercalation reactions in liquid ammonia is the introduction of alkaline-earth metals into layered nitride chlorides [6]. In β-HfNCl, alkaline-earth metals such as Ca, Sr, and Ba can be dissolved in liquid ammonia and subsequently intercalated into the van der Waals gap of the layered host structure. The resulting compounds *AE_x_*(NH_3_)*_y_*HfNCl exhibit superconductivity with *T*_c_ values typically in the range of 23–24 K over a wide range of electron-doping concentrations. The basal spacing of the ammonia-cointercalated compounds is typically around 12 Å, reflecting the presence of ammonia molecules between the layers. Furthermore, co-intercalation of larger solvent molecules, such as tetrahydrofuran (THF), can expand the interlayer spacing and increase the *T*_c_ to about 26 K [6].

This liquid-ammonia intercalation approach is not limited to alkaline-earth metals and can also be extended to other electron donors. For example, rare-earth metals such as Eu and Yb have been successfully introduced into β-*M*NCl (*M* = Zr, Hf) using metal–ammonia solutions [72]. The resulting compounds *RE_x_*(NH_3_)*_y_M*NCl also exhibit superconductivity with transition temperatures comparable to those of alkali-metal intercalated analogues.

These studies demonstrate that liquid-ammonia solutions provide a powerful and versatile reaction medium for electron doping and intercalation in layered nitride halides, enabling systematic control over their electronic structures and superconducting properties.

Practical considerations. Liquid-ammonia-based reactions provide a flexible route for intercalation and precursor formation, typically carried out within several hours at low temperatures. The reaction scale can be adjusted according to the size of the reaction vessel, allowing moderate-scale synthesis when required. However, the method requires specialised handling of liquefied ammonia, including low-temperature operation and appropriate precautions due to its toxicity and cryogenic nature.

## 4. Hybrid Gas–Liquid Ammonia Reactions

Hybrid gas-liquid ammonia reaction systems can be constructed by coupling a liquid-ammonia reservoir with a high-temperature tube furnace via metal tubing. This hybrid approach bridges liquid-phase and gas-phase ammonia chemistry, providing a unified platform for controlling reaction pathways across a wide temperature range. Although the subsequent high-temperature reaction itself proceeds entirely in the gas phase, the system is classified as hybrid because ammonia is first purified in the liquid phase using dissolved alkali metal and then supplied to the reaction zone as a gas. This liquid-phase purification step is essential for achieving a super-high-purity NH_3_ atmosphere.

### 4.1. Super-High-Purity NH_3_ Gas Reaction System

**Figure 7:**
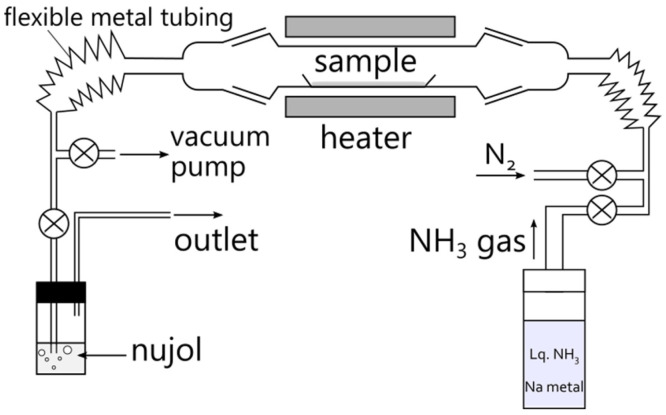
Schematic illustration of a hybrid gas–liquid ammonia reaction system for enabling a continuous flow of super-high-purity NH_3_ gas for high-temperature reactions. (Adapted from Ref. [7] (CC BY 4.0)).

Based on this concept, a horizontal tube furnace system equipped with an evacuable quartz reaction tube is connected to a liquid-ammonia reservoir via metal tubing, as illustrated in Figure 7. This configuration enables high-temperature reactions under a continuous flow of super-high-purity NH_3_ gas [7].

Such a setup is critically important for reactions involving transition metals that are highly susceptible to oxidation at elevated temperatures, including Ti, Zr, and Hf. In these systems, even trace amounts of oxygen or moisture can lead to the formation of oxide and oxynitride phases, which significantly deteriorate the product quality, as demonstrated in previous studies on zirconium oxynitrides and related systems [73,74].

In a typical procedure, the sample is placed at the centre of a quartz reaction tube, and the system is repeatedly purged with an inert gas (e.g., N_2_) to remove residual air. The gas flow is then switched to purified NH_3_, allowing the sample to be heated up to ~1000 °C under a continuous ammonia flow. Although this configuration can, in principle, be adapted to other gas atmospheres, the present review focuses on ammonia-mediated reactions.

**Figure 8:**
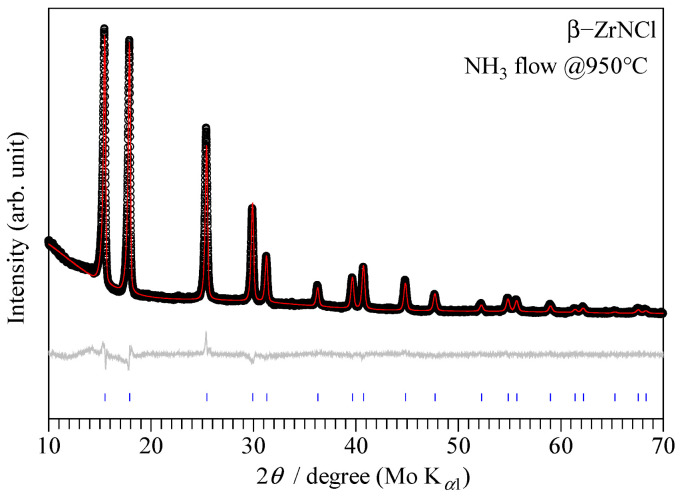
Profile plots from the Rietveld refinements against X-ray powder diffraction data for the products of the reaction of β-ZrNCl under streams of flowing NH_3_ (at 950 °C). Open circles denote the observed data points, and the solid red line represents the calculated diffraction pattern. The difference profile is in grey, and the phase markers for the ZrN phase are indicated in blue. (Adapted from Ref. [7] (CC BY 4.0)).

As a representative example, β-ZrNCl can be converted into nitrogen-rich ZrN phases via ammonothermal dechlorination under flowing ammonia gas. The X-ray diffraction profile of the product obtained at 950 °C is shown in Figure 8. Despite the well-known tendency of zirconium compounds to oxidise readily at high temperatures, no significant oxide-related peaks are observed, even after prolonged high-temperature treatment, demonstrating the effective suppression of oxidation under these conditions. This result clearly demonstrates that the present system provides an exceptionally clean reaction environment, enabling high-temperature nitridation processes that are otherwise difficult to achieve because of oxidation.

### 4.2. Formation of N-Rich ZrN Derived from MNX

**Figure 9:**
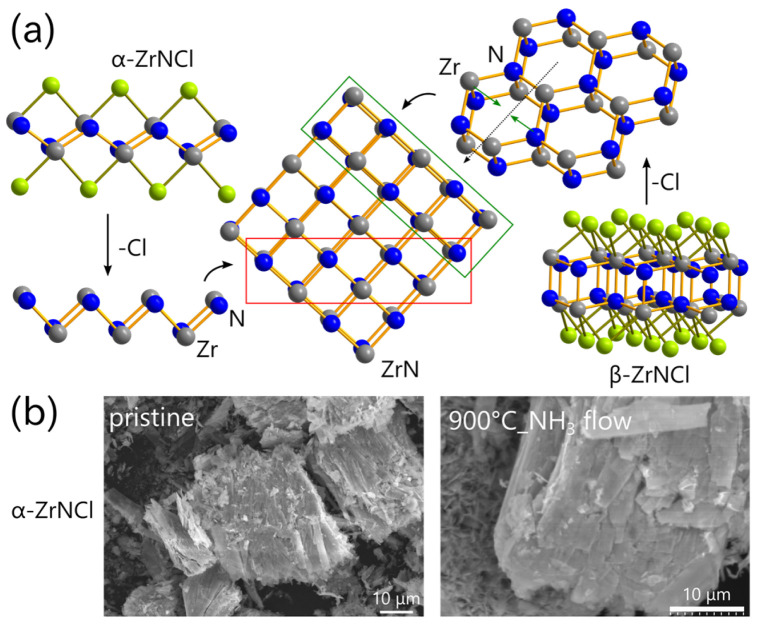
(**a**) Schematic illustration of the rock-salt structure of ZrN (**centre**) as derived from the α- (**left**) and β-polymorphic structures (**right**) of ZrNCl. (**b**) SEM images of zirconium nitride obtained by ammonothermal dechlorination of α-ZrNCl under the NH_3_ stream. The ZrN appears to retain the original morphology of the starting polymorph (Adapted from Ref. [7] (CC BY 4.0)).

The dechlorination reaction described above can be further rationalised from a structural viewpoint, as illustrated in Figure 9. In the context of this review, this transformation is considered an extension of ammonia-mediated synthesis of “layered nitride” materials, as it derives new nitride phases from layered precursors. Both α- and β-form ZrNCl consist of layered structures in which Zr–N networks are separated by halogen layers. Upon chlorine removal, these layered frameworks can be transformed into rock-salt-type ZrN while partially preserving their two-dimensional structural morphology. This transformation provides a useful route to bulk synthesis of ZrN with a layered morphology.

Although the reaction shown in Figure 8 also falls within this category, it is instructive to explicitly consider the structural relationship. In particular, when starting from the α-form polymorph, the layered nature of the precursor is more directly reflected in the resulting morphology, as shown in Figure 9b. The obtained ZrN retains features of the original layered structure, indicating that the dechlorination proceeds via a topochemical-like transformation. This approach therefore offers a pathway not only for synthesising ZrN but also for controlling its microstructure by selecting the precursor polymorph [7].

Further insight into the composition of the obtained ZrN can be obtained from thermal analysis. Temperature-programmed desorption measurements confirm that nitrogen is released upon subsequent heating, indicating that the as-prepared samples contain excess nitrogen, even though the lattice constant shrinks after the reheating. By carefully selecting the reaction conditions under flowing NH_3_, the nitrogen content can be systematically controlled. In particular, when the reaction is initiated from the α-type polymorph, the resulting ZrN exhibits the highest N/Zr ratio, approaching that of Zr_3_N_4_ stoichiometry while retaining the rock-salt-type framework. Notably, the nitrogen-rich composition is accommodated primarily through Zr deficiency rather than interstitial nitrogen. This represents the first example of bulk synthesis of nitrogen-rich ZrN. Such a defect mechanism is highly unusual for transition metal nitrides and highlights the distinct reaction pathway enabled by ammonia-mediated processes.

**Figure 10:**
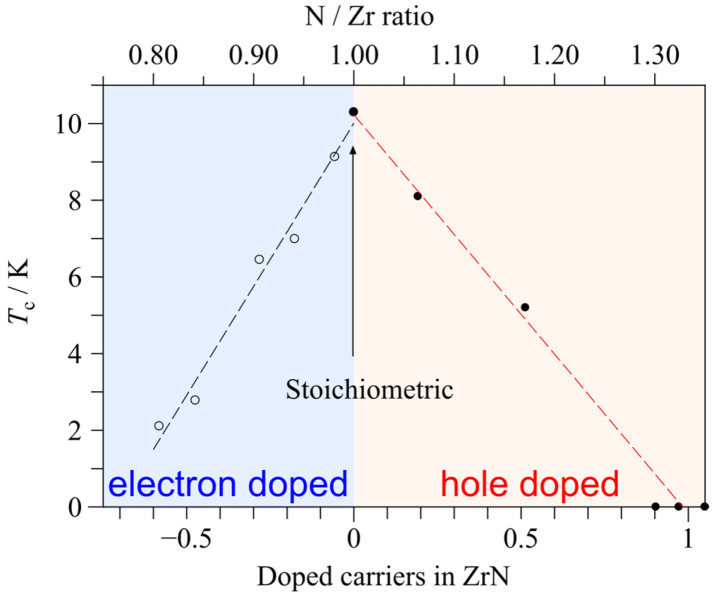
Dependence of *T*_c_ on N:Zr ratio and carrier concentration in rock-salt zirconium nitrides. The “Stoichiometric” point (1.00) corresponds to the commercial reagent ZrN. Data points shown in open circles in the electron-doped region were taken from Ref. [77]. (Adapted from ref. [7] (CC BY 4.0)).

The superconducting properties of the obtained ZrN are summarised in Figure 10. Zirconium nitride is known as a conventional superconductor at low temperatures, and the present nitrogen-rich ZrN also exhibits superconductivity in the nitrogen-excess regime. Because the nitrogen-rich composition is associated with Zr deficiency, the carrier concentration can be tuned through the vacancy concentration generated during synthesis. Accordingly, *T*_c_ shows a dome-like dependence on the nitrogen content. Such dome-shaped behaviour is typically observed only in a limited class of superconductors with relatively high transition temperatures, such as cuprates and iron-based superconductors [75,76]. In this system, both electron- and hole-doping can be effectively tuned through composition, providing a rare example in which carrier concentration is controlled in a simple binary nitride. The emergence of these properties in a well-known and industrially important material such as ZrN is uncommon. These results demonstrate that combining ammonia-mediated synthesis with appropriate precursor selection enables access to previously unexplored compositional and electronic states, even in well-established conventional nitride materials.

Practical considerations. Hybrid gas–liquid ammonia systems enable high-temperature reactions under extremely clean conditions, which are particularly advantageous for oxygen-sensitive materials. Once established, the reactions themselves are relatively straightforward, although the overall system requires greater technical complexity, including ammonia purification, metal tubing, and vacuum-compatible connections. These methods are therefore best suited for advanced studies where precise control of the reaction atmosphere is essential.

## 5. Conclusions

In this review, we summarised ammonia-mediated synthetic routes for layered nitride halides and related nitride materials, with particular emphasis on selected *M*N*X* systems, especially Ti- and Zr-based chlorides and iodides. Ammonia plays multiple roles in these reactions, functioning not only as a nitrogen source but also as a reactive environment that enables ammonolysis, intercalation, and defect formation.

Gas-phase reactions provide a fundamental route to *M*N*X* formation, in which ammonolysis of metal halides followed by ammonia-assisted chemical vapour transport enables the synthesis of highly crystalline layered nitrides. This combination of ammonolysis and transport processes has been established as a key strategy for obtaining phase-pure *M*N*X* materials.

Liquid ammonia further extends the scope of ammonia chemistry by enabling intercalation reactions. These reactions provide an effective route for introducing guest species into layered host materials, allowing systematic control of carrier concentration and structural modification, leading to superconductivity and other emergent properties.

By integrating these approaches, hybrid gas–liquid ammonia reaction systems provide a unique and powerful platform for high-temperature reactions under extremely clean conditions. This methodology is particularly important for oxygen-sensitive transition-metal systems, where oxidation must be suppressed to access intrinsic material properties.

As demonstrated in the case of ZrN derived from layered precursors, ammonia-mediated reactions enable not only phase transformation but also precise control of composition and defect chemistry. The stabilisation of nitrogen-rich ZrN through zirconium deficiency, together with the emergence of dome-shaped superconducting behaviour, highlights the potential of ammonia chemistry to access previously unexplored electronic states, even in well-established materials.

These findings underscore the versatility of ammonia as a reaction medium for both synthesis and materials design. More broadly, ammonia-mediated processes provide a unified framework for controlling reaction pathways across gas, liquid, and hybrid environments. The combination of such approaches with appropriate precursor selection offers a promising route towards the discovery of new functional materials and the re-examination of conventional systems from a fresh perspective.

## Figures and Tables

**Table 1:** Lattice parameters, selected bond distances, angles of TiNI and related compounds.

	TiN [14]	TiNCl [14]	TiNI [11]	TiNI (This work)
Spacegroup	*Fm-3m* (no. 225)	*Pmmn* (no. 59)		*Pmmn* (no. 59)
*Z*	4	2		2
Lattice parameters/Å				
*a*	4.244	3.9382(1)	3.941 ^1^	3.9401(2)
*b*		3.2582(1)	3.515 ^1^	3.5162(2)
*c*		7.8001(1)	8.955 ^1^	8.9449(4)
Selected bond distances (Å) and angles (°)				
Ti-N (i)	2.122	2.0077(8)		1.996(4)
Ti-N (ii)	2.122	2.0145(23)		2.034(2)
Ti-X		2.4299(12)		2.771(2)
Ti-Ti	3.001	3.0029(8)		3.0135(12)
N-Ti-N (α)	180	157.50(1)		151.126(2)
N-Ti-N, Ti-N-Ti (β)	90	107.93(1)		123.459(1)

^1^ No standard deviations available.

**Table 2:** Atomic coordinates and occupancy parameters of TiNI determined in this work.

TiNI (This work)					
Atom (site)	*x*	*y*	*z*	*Occ*	*B*/Å^2^
Ti (2b)	0	1/2	0.0812(2)	1	1
N (2a)	1/2	1/2	0.0245(8)	1	1
I (2a)	0	0	0.32061(9)	0.917(4)	1
*R_wp_, R_exp_* (%)	2.049, 3.111				

## Data Availability

The data supporting the findings of this study are included within the article. Additional details are available from the corresponding author upon reasonable request.
